# The Intimate Structure of the Axis-Cylinder and of Nerve-Cells

**Published:** 1869-08-15

**Authors:** 

**Affiliations:** Liege


### CHICAGO MEDICAL JOURNAL.

VOL. XXVI.—AUGUST 15, 1869.—No. 16.

### TRANSLATIONS.

### *The Intimate Structure of the Axis-Cylinder and of Nerve-Cells.*

BY DOCTOR GRANDRY, (OF LIEGE).

*Preliminary Remarks.*

The history of the nervous system has been made the object of numerous researches, of late years; but the greater part of the works have treated rather of the relative dispositions of the different nerve-elements in the centres, and of their different modes of termination, than of the intimate structure of the fundamental elements. Remak, however, since the discovery of the axis-cylinder, had already pointed out the longitudinal striation of this element, a striation which he believed to exist at the periphery. The use of re-agents acting upon the nerve-elements, either in an especial manner or by preserving them in a state of integrity more or less perfect, has permitted the unveiling of a structure peculiar to the fundamental elements of the nervous system.

M. Shultze,* by making use of iodo-serum, of the cephalo-rachidian liquid, of the bichromate of potassa in solution in water (from ½ to 1 per cent.), of chromic acid (from 1-40 to 1-20 per cent.), of hyperosmic acid (from ½ to 1 per cent.), has succeeded in demonstrating that a great number of very fine fibrillæ are found in the interior of the nerve-cells of the electric lobes of the torpedo, of the spinal cord and of the cortical substance of the cerebrum and cerebellum. The fibrillæ spread themselves without regular order in the interior of the cells, but this author has not been able to discover their mode of termination in the nerve-cell. He has always seen them losing themselves in the granular substance, but never in the nucleus. The fibrillæ are very fine; some of them, however, attain the size of $0.0005^{mm}$.

The prolongations of the cells are thus seen formed by fibrillæ separated from each other by interposed granular matter. It is possible, especially in the prolongations of the cells, to isolate the fibrillæ by maceration in the solution of bichromate of potassa. According to M. Shultze, the axis-cylinder should have the same composition as the prolongation, and the longitudinal striation already determined by Remak would appear not only in the periphery but throughout the entire thickness of the axis-cylinder. M. Schultze concludes the axis-cylinder and the nerve-cells can not be considered as the ultimate elements of the nervous system, but that these elements are themselves formed from more minute elements, primitive nerve-fibrillæ connected together by an intermediate granular substance.

Other authors had already indicated the existence of fibrillæ in nerve-cells, Arnold and Courvoisier, especially, in the ganglion-cells of the sympathetic nerve of the frog.

Fromman,† by the use of very slightly diluted albumen, discovered fibrillæ in the interior of the nerve-cells of the anterior cornua of the spinal cord of the ox; but according

* Discours Académique, Bonn., Aout, 1868.

† Virchow's Archiv., t. XXXI.

to this author, contrary to the opinion of Max Schultze, the fibrillæ should have intimate connections with the nuclei and the nucleoli. The same author, using nitrate of silver as a medium of impregnations, arrived at the following results: "In the greater number of cases the axis-cylinder, whatever may be its volume, presents a transverse striation; sometimes a longitudinal striation is recognized. The nerve-cells, treated in the same manner, are exhibited with a black nucleolus, a colorless nucleus, and the body of the cell colored; upon the prolongations, by the use of albumen, the longitudinal striæ already referred to may be discovered."

The results which I shall exhibit correspond perfectly with those of Fromman, as to the axis-cylinder, but I arrive at very different conclusions as to the re-actions presented by the nerve-cells. This difference of opinion being probably occasioned by the different methods employed, I shall describe in detail the manner in which I proceeded in my investigations. Fromman, moreover, in his work does not specify exactly in every point his mode of procedure.

### MEDIA OF STUDY.

My investigations have been made upon the ganglion of Gasser and the spinal cord, the cerebrum and the cerebellum of the ox, the great sympathetic and sciatic nerve of the frog. M. Legros, who has repeated my investigations, in the laboratory of the Faculty of Medicine, Paris, arrived at the same results as I, experimenting upon the same organs in the rabbit.

The organs have invariably been removed as soon as possible after death (on the average half an hour after), so that they were still living, or that at least they might be supposed not to have undergone any sensible cadaveric alteration.

This circumstance is indispensable; the same results cannot be attained with the spinal cords of animals dead more than six hours.

As soon as removed the organs were plunged into a solution of crystallized nitrate of silver of 1 to 400, after having previously been cut into fragments of 1 to 1½ centimetres. This accomplished, I placed them in a receptacle excluded from the light, and at the ordinary temperature, during about five days; then I exposed them to the light during two or three days, leaving them in the silver solution in which they were macerating. This period of action of the re-agent and of light is in general sufficient, but the results appeared to be better if they were permitted to act a longer time; some of my most beautiful preparations have been obtained after fifteen days of the action of the nitrate of silver in darkness and about the same period of exposure to the light.

I should remark that these results were obtained by exposing the objcts to a low temperature, but that in summer the decomposition of the elements occurs on the fourth day. The preparations obtained after fifteen days of maceration were very beautiful at first, but at the end of three or four months they were completely altered.

The silver solution which has succeeded best is that of one four-hundredth. More concentrated solutions gave less satisfactory results. Fromman employed a solution of one six-hundredth for the centres, and one three-hundredth for the peripheral nerves.

The quality of the light has great influence; it is much preferable to use very intense light; it gives much more beautiful results, after a relatively brief period of action, than a feebler light acting for a longer time. In the sunlight sometimes less than half an hour is sufficient to render apparent the re-action in the axis-cylinders.

The action of light is perceptible only upon fragments; hence it follows that this portion only can be used for study; however, the rest is not lost, because the nitrate of silver acts to the depth of two or three millimetres, so that by making sections perpendicular to the surface, and exposing them to the light, a new series of preparations sufficient for the study of the axis-cylinder may be obtained.

In order to make the preparation, it is sufficient to remove a portion of the part which it is desired to examine, by the aid of scissors, or by scraping the surface with the back of a scalpel; the particles thus obtained should be placed upon the slide in a drop of glycerine, either pure or mingled with a little acetic acid, and distended as much as possible.

Instead of making soft preparations, alcohol may be used in order to harden them, and thin slices made and placed in Canada Balsam; the same result may be obtained thus as by proceeding in the manner previously described.

It is absolutely indispensable to follow exactly the process which I have just described in order to obtain, with any degree of certainty, the results detailed above. Failure in the re-actions has sometimes happened to me, but this was always due to the fact that I did not follow vigorously this mode of procedure.

### THE AXIS-CYLINDER.

The axis-cylinder should be studied under two conditions: 1st. When it is free, not included in the interior of a fibre, and consequently in immediate contact with the re-agent; 2d. Included in the interior of a fibre, and enveloped by the cell-less sheathe and the cord. I shall commence by studying it in the free state.

The organ most suitable for this investigation is the spinal cord, although it may be as readily made in the ganglion of Gasser, but this latter is better adapted to its investigation in the complete fibre.

The organ having been prepared as I have already described, it will be seen immediately, even when slightly magnified, that the principal and characteristic re-action of the axis-cylinder, whatever may be its volume, is a very marked and very regular transversal striation, similar to that of striated muscles. Throughout its entire extent the cylinder presents portions alternately clear and dark, as if the re-agent had attacked one portion of the substance and spared the other.

It will, likewise, at once be seen that the opaque striæ are found in a definite portion, at regular distances, but that these distances as well as the thickness of the striæ themselves vary in different portions.

The thickness of the striæ varies from $0.001^{mm}$ to $0.005^{mm}$; that of the spaces which separate them from $0.001^{mm}$ to $0.003^{mm}$ approximately.*

Considered separately, each of the striæ is seen in the form of a line more or less thick, uniformly colored in the finest, more or less granular and less clearly defined in the larger. This last fact is not constant, for frequently the large striæ are found perfectly homogeneous. If, while observing, the focus of the objective be varied, it will be seen, and this is more readily observed in striæ of medium size, that the stripe which had the appearance of a line uniformly colored, becomes clearer at its centre, and is sharply defined by a deeply colored contour which presents a rectangular form with rounded outlines.

The matter interposed between the striæ is ordinarily lightly colored, but much less so than the striæ; sometimes it is altogether colorless; this depends upon the more or less prolonged action of the re-agent and of the light. It is more abundant between the large than between the fine stripes, but it happens, however, that the large stripes are very closely approximated to each other.

The stripes, whatever their thickness, bear the same relation to each other; they have all the same dimensions in a certain extent and are regularly spaced. As to their direction, they are all placed perpendicularly to the axis of the cylinder, when that is rectilinear; when it is curved, if the curve is slight, the curves radiate from the centre, without changing their form; but in the case of a sharp curve, they change their form, and from rectangular they become triangular, the summits of the triangles being directed to the centre of the curvature.

---

*The spaces between the striæ of the muscles are from $0.0009^{mm}$ to $0.002^{mm}$.

The stripes vary in thickness. It might be supposed that the finest would be found in the smallest axis-cylinders and *vice versa.* It is not so, however. No relation exists between the thickness of the stripes and the volume of the cylinder, for the finest stripes are sometimes found in the largest cylinders.

If a cylinder be examined over a great extent, it will be seen that while the stripes havethes ame thickness, this, likewise, is variable. In this latter case we may find an insensible transition; but most frequently it is abrupt, and then there is found between the two kinds of stripes a deeply colored space, larger than the largest stripes.

There are frequently found upon the same cylinder several of these deeply colored spaces which separate groups of stripes either of the same or of different thickness. All that I have just described is very manifest in cylinders well stained, but it frequently happens that the action of the reagent is too strong or too feeble. In case the action be too strong, the stripes are sometimes seen indistinctly, but ordinarily the entire cylinder presents a uniform deep brown color. When the re-action has been too feeble the stripes are granular, punctuated; but it frequently happens that stripes are found clearly marked from space to space. Sometimes the only effect obtained consists in the presence of blackish granulations, disseminated irregularly upon the cylinder.

Such are the characteristics presented by the axis-cylinder when placed, still living, in a solution of nitrate of silver, and subsequently exposed to the action of light.

Besides the transverse, Fromman speaks of longitudinal stripes, but he does not designate the conditions under which these were recognized. I, also, have detected them, but in cords which had been plunged into nitrate of silver, not at the ordinary temperature, but had been, in the meantime, submitted to a temperature below zero. These longitudinal stripes, although quite plainly visible, are much less marked than the transverse.

I have likewise detected the transverse stripes by my process, though rarely. I have even seen, at times, the axis-cylinder, and especially the prolongations of the cells, presenting the two orders of stripes simultaneously, so that the entire surface presented a well-marked check.

The preceding remarks apply to the axis-cylinder completely isolated. Let us now see how it will behave in the interior of a complete fibre, when it is placed under the same conditions.

If an isolated cylinder be traced into the interior of a fibre, it will be seen that the stripes exist, not only upon the denuded part but likewise upon that which is enveloped by the acellular sheathe and the cord, and that, over a certain extent they are marked, as well marked then as upon the outside of the fibre; if the cylinder be traced farther the stripes will soon be seen to become less marked, dotted, and then to be no longer manifest. The action of the re-agent is only manifest near the points where the axis-cylinder becomes free, and dimihsshes in intensity in proportion as it (the axis-cylinder) penetrates into the interior of the fibre.

This is due, probably, to the fact that the nitrate of silver has penetrated to a certain depth by gradual imbibition, and that this imbibition not being possible farther through the acellular sheathe and the cord, the re-agent can no longer act.

It has been already observed that all the stripes are parallel and separated from each other by a matter upon which the nitrate of silver acts feebly.

What is the effect of compression upon this condition? The compression may be vertical, either accompanied by lateral movements or not. Simple vertical compression has no effect upon the finest stripes; the largest expand as well as do the spaces which separate them. If pressure with lateral motion be brought to bear upon the stripes of medium size, well tinted, and especially upon cylinders not very voluminous, sometimes one and sometimes several stripes will frequently be seen to deviate from the common direction. I have seen stripes displaced singly, without change of form, and assume positions relatively oblique to the others, and even to be placed perpendicular to them, that is, in the direction of the axis-cylinder, just as if this latter were formed of resistant discs separated by a softer substance, some of which might be pressed down obliquely or longitudinally. By the same means it happens, frequently that nearly all the stripes may deviate irregularly from their normal position, in every direction, these varieties of position preserving no relation to the curvature of the cylinder.

The partial rupture of the cylinders may also be effected; and then it will be seen that this occurs in the intervals between the stripes, these latter remaining perfectly intact.

Whatever may be the degree of compression employed, it is very difficult to destroy the stripes, even when they are granular. The effect of the pressure is manifested upon the interposed matter which is much less resistant than the stripes.

Besides the displacement of the stripes, their complete isolation may be effected. In order to accomplish this, I used preparations which had remained a considerable time in glycerine, and applied to the object strong pressure with lateral movements. To reach the same end, it is as well to use fragments of organs macerated several weeks in the silver solution after exposure to the action of light, and to proceed as above in making the preparation with glycerine. By proceeding thus, the stripes may be isolated with facility, and will be seen to present the same characteristics as when they are re-united; generally it is difficult to free them completely from the interposed matter of which a very small quantity remains adherent.

From the facts thus set forth it may be concluded:

1st. That nitrate of silver has a special action upon certain portions of the axis-cylinder, and affect certain others but feebly.

2d. The axis-cylinder is composed of two substances differing by their chemical and physical properties.

3d. These two substances are not mingled, but completely isolated, and present an arrangement regular by relation to each other.

4th. The axis-cylinder is formed of superposed discs separated from each other by a substance which has not the same properties as the discs themselves.

5th. The axis-cylinder contained within the interior of a complete fibre, offers absolutely the same characteristics as the isolated cylinder, in so far as the re-agent can act upon the cylinder itself.

In the examining the axis-cylinder striped transversely, the resemblance which it presents to striated muscular fibre is striking. The characteristics common to muscles and nerves are now much more clearly defined, since M. Schultze has isolated the longitudinal fibrillæ. And here is presented the same discussion as in reference to muscles, that is to say whether the discs or the fibrillæ constitute the ultimate element of the axis-cylinder. I should remark, however, that the axis-cylinder does not behave as do muscles under polarized light. New researches ought still to be established in order to determine rigorously the fundamental element of the axis-cylinder.

### NERVE-CELLS AND THEIR PROLONGATIONS.

Before commencing the exposition of my investigations of nerve-cells, I deem it useful to recall, in a few words, the preparation to which the object must be submitted, because it is especially in this particular that my results disagree with those of Fromman, which disagreement I attribute to the difference in the conditions in which we were placed.

The organs were taken as *much alive* as possible, plunged for a certain time into a solution of nitrate of silver, then exposed to the light; upon nerve-cells it is important to permit light to act for a long time, because its action upon them is much weaker than upon the axis-cylinders.

The organ upon which the best re-ults are obtained is the spinal cord (cervical region), and it should also be stated that the observations which follow were made principally upon the cells of the anterior cornua.

Fromman, acting with nitrate of silver upon congealed cords, found the bodies of the cells uniformly colored brown, the nucleus remaining colorless; the prolongations presenting longitudinal, but not transverse stripes.

If the organ be prepared as I have indicated, it will be perceived that, as in the case of the axis-cylinder, the action of the nitrate of silver occasions a very decided striation of the body of the cell and of its prolongations; parts are seen alternately clear and dark.

The striæ vary in thickness from 0.001 mm. to 0.005 mm.

The finest stripes are seen to be completely homogeneous, of a brown color; the largest are generally dotted, granular, as though formed of blackish granulations aggregated together. Between the striæ is found a substance less tinted, sometimes altogether colorless. A cell may present only striæ of the same thickness, but frequently upon the same cell, as upon the axis cylinder, are found striæ of variable thickness. The cells present, likewise, spaces deeply colored, larger than the ordinary striæ, and are thus divided into two or three segments with stripes of the same or different thickness. As to the direction of the stripes no rule can be given. They are all parallel; the nucleus has no influence upon them; they pass above it.

The cells are not always attacked by the re-agent over their whole surface; then, in this case, the cell is seen transversed, so to speak, cut into two by a colored plane which terminates at the surface by a stripe.

By placing myself under the conditions indicated by Fromman I have, as has this author, seen the body of the cell colored uniformly brown, and the nucleus remaining intact; at the same time I have recognized the longitudinal stripes upon the prolongations, but my observations did not permit me to decide upon their mode of termination in the interior of the cell.

The prolongation of the cells present a transverse striation, in all respects similar to that of the axis-cylinder; as to the thickness of the stripes, it corresponds to that of the stripes of the segment of the cell from which they originate. This is seen in the entire cells, and especially in the prolongations which, torn off, have dragged with them parts of the cell.

If a prolongation be curved around the cell, the striation is not perceptible, but if such movements be imparted to the preparation as to render it rectilinear, immediately the stripes are exhibited very clearly defined. The prolongation becoming re-curved the striation again disappears.

If the nerve cells be compressed, an enlargement of the large stripes is effected, the finer ones undergoing no change. Compression associated with lateral movements produces no isolation of the stripes as in the case of the axis-cylinder; the only effect which can be obtained is to render the stripes sinuous, although leaving them parallel; it should be observed, however, that the stripes can only be destroyed with great difficulty.

Besides the transverse striation the double striation can be detected in the nerve-cells as in the axis-cylinder; in this case I have observed no relation of the longitudinal stripes with the nucleus.

The nerve-cells in which I have clearly determined the striation are those of the ganglion of Gasser; of the anterior cornua of the spinal cord, and of the floor of the fourth ventricle.

From these facts it may be concluded, that

1st. Two substances differing by their properties exist in the body of the cell.

2d. These two substances appear to be chemically isolated.

3d. There is, perhaps, an arrangement in discs, as in the axis-cylinder; but the only fact in evidence is the existence, in certain cases, of a colored plane cutting the cell entirely.

4th. The axis-cylinder and the nerve-cells exhibit the same characteristics when they are submitted, under certain conditions, to the action of nitrate of silver

In consequence of the reactions produced by nitrate of silver upon the axis-cylinder and nerve-cells, we may hope to elucidate the questions of the connective tissue and of the nerve-substance in the posterior cornua of the spinal cord and elsewhere. The investigations which constitute the subject of this memoir were conducted in the laboratory of the Faculty of Medécine of Liege. I must here express my gratitude to Professor Schwann, for the kind feeling which he has manifested toward me in guiding me in my labors.

The work was presented to the Academy of Sciences of Belgium, and we subjoin the conclusions of Professor Schwann.*

"The work of M. Grandry concerns the most important elementary parts of the body. The discussions which they have originated will doubtless revolve around the question whether the striæ seen in the axis-cylinder and the nerve cells, after the use of nitrate of silver upon these organs, still alive, pre-exist before the re-agent, or whether they are artificial products.

"It appears to me difficult to admit that formations so regular, as the stripes in question, *could* be obtained artificially, if there was not already in the living organ a corresponding arrangement. It is not, however, necessary to admit the discontinuity of the discs. There is, perhaps, a homogeneous substance in which are deposited molecules of another sort susceptible to the action of nitrate of silver. These molecules would impregnate certain layers, hardening them, and would leave other layers free. W. H.

---

* *Bulletin de l'Academie des Sciences Belgique.* 87th annee, 2d Serie, XXV, p. 287.

